# Vaccination against Typhoid Fever

**Published:** 1897-10-30

**Authors:** 


### VACCINATION AGAINST TYPHOID FEVER.

Currency was given some time ago to a statement that a quantity of the material which has been devised as a "vaccine" against typhoid fever had been taken to Maidstone by Surgeon-Major Semple, the supply, in fact, being ample, and "enough for the whole population" of the town. A few words, then, on the nature and possible efficacy of this material—this so-called "vaccine"—will not be out of place.

Starting with the well-known fact that the specific fevers confer upon those who suffer from them a varying degree of immunity from further attack, lasting in many cases for life, we find that a similar immunity can be produced by inoculation with the micro-organism to which the disease is due. This, however, is, to all intents and purposes, a matter of giving the disease itself, and cannot be called a vaccination. A certain immunity can, however, be produced by the use of micro-organisms whose virulence has been attenuated—naturally by being passed through other animals, as in true vaccination—or artifically in various ways. Again, immunity can be produced by the injection of a certain quantity of dead, but still poisonous, micro-organism, or, in other words, of the products of the micro-organisms. Speaking roughly, we may put it thus: disease is caused not directly by the infective micro-organisms, but by their products—toxins—and these substances stimulate the tissues to produce other substances—anti-toxins—by which their toxic effects are neutralised, and immunity is produced. Immunity, then, may either be produced by the stimulating and irritating action of microbes and their products within the body, or may be acquired by the injection of quantities of the poisonous products of microbes which themselves have been grown outside and then have been killed. Such is the theory. The last process mentioned is the basis of the anti-plague inoculation of Haffkine, and the anti-typhoid "vaccination" of Wright and Semple. A cultivation of typhoid bacilli is made. It is standardised by experiment on guinea-pigs of a given weight, and then the bacilli are killed by exposure for a given time to a given heat. This is the "vaccine"—a culture containing the dead bacilli and all their products.

Now we come to another side of the story. The blood of a patient who is undergoing or has undergone typhoid fever produces in a cultivation of typhoid bacilli certain changes, among which what has been described as agglutination or "sedimentation" is the most obvious. This is the essence of what is spoken of as Widal's test. At last we come to the point. The injection of the anti-typhoid "vaccine" certainly produces in the blood of the patient the same power of "sedimentation" as is produced by undergoing the fever, but does it give the same immunity?

The only answer that can at present be given to this question appears to be that guinea-pigs and monkeys which have been "vaccinated" by the material described, and whose blood has developed this property of "sedimentation," have also shown themselves extremely resistant to infection by the micro-organisms in question.

The vaccination then produces the same condition of blood as is found in persons rendered immune by an attack of typhoid fever, and animals in whom this condition has been produced by vaccination have been found to possess a certain "bacteria proofness." Whether such "bacteria proofness" is produced in man by vaccination, and whether that condition involves immunity to the disease when the infection enters by the natural channels, are questions yet to be solved. Unfortunately, it does not seem likely that the use of this method at Maidstone will enlighten us greatly. If a large section of the inhabitants of some town or village with a fairly high, steady typhoid rate were to be vaccinated, while other conditions were left unchanged, some useful statistical results might be obtained, but it does not seem to us likely that any reliable data will be gained from the vaccination of any number of people during the swing of an epidemic, especially of one which is so rapidly on the wane.

